# Changes in Underlying Determinants Explain Rapid Increases in Child Linear Growth in Alive & Thrive Study Areas between 2010 and 2014 in Bangladesh and Vietnam^1^,^2^,^3^

**DOI:** 10.3945/jn.116.243949

**Published:** 2017-01-25

**Authors:** Phuong Hong Nguyen, Derek Headey, Edward A Frongillo, Lan Mai Tran, Rahul Rawat, Marie T Ruel, Purnima Menon

**Affiliations:** 4 Poverty, Health, and Nutrition Division, International Food Policy Research Institute, Washington, DC;; 5 Department of Health Promotion, Education, and Behavior, University of South Carolina, Columbia, SC; and; 6 FHI 360, Hanoi, Vietnam

**Keywords:** decomposition, HAZ, linear growth, Bangladesh, Vietnam, underlying determinants

## Abstract

**Background:** Child linear growth sometimes improves in both intervention and comparison groups in evaluations of nutrition interventions, possibly because of spillover intervention effects to nonintervention areas or improvements in underlying determinants of nutritional change in both areas.

**Objective:** We aimed to understand what changes in underlying socioeconomic characteristics and behavioral factors are important in explaining improvements in child linear growth.

**Methods:** Baseline (2010) and endline (2014) surveys from the Alive & Thrive impact evaluation were used to identify the underlying determinants of height-for-age *z* scores (HAZs) among children aged 24–48 mo in Bangladesh (*n* = 4311) and 24–59 mo in Vietnam (*n* = 4002). Oaxaca-Blinder regression decompositions were used to examine which underlying determinants contributed to HAZ changes over time.

**Results:** HAZs improved significantly between 2010 and 2014 in Bangladesh (∼0.18 SDs) and Vietnam (0.25 SDs). Underlying determinants improved substantially over time and were larger in Vietnam than in Bangladesh. Multiple regression models revealed significant associations between changes in HAZs and socioeconomic status (SES), food security, maternal education, hygiene, and birth weight in both countries. Changes in HAZs were significantly associated with maternal nutrition knowledge and child dietary diversity in Bangladesh, and with prenatal visits in Vietnam. Improvements in maternal nutrition knowledge in Bangladesh accounted for 20% of the total HAZ change, followed by maternal education (13%), SES (12%), hygiene (10%), and food security (9%). HAZ improvements in Vietnam were accounted for by changes in SES (26%), prenatal visits (25%), hygiene (19%), child birth weight (10%), and maternal education (7%). The decomposition models in both countries performed well, explaining >75% of the HAZ changes.

**Conclusions:** Decomposition is a useful and simple technique for analyzing nonintervention drivers of nutritional change in intervention and comparison areas. Improvements in underlying determinants explained rapid improvements in HAZs between 2010 and 2014 in Bangladesh and Vietnam.

## Introduction

Child undernutrition is a substantial public health concern in developing countries. In 2014, ∼23.8% of children aged <5 y were stunted, reflecting a 15.8% decline from 39.6% since 1990 (1). Stunting has numerous short- and long-term consequences, including increased childhood morbidity and mortality (2, 3), delayed gross and motor development (4), and long-term educational and economic consequences (5).

A set of nutrition-specific interventions of known efficacy has been identified to prevent stunting in young children (6). Key challenges occur, however, when evaluating the impact of these interventions under real-life programmatic conditions. One of these challenges, especially when programs are evaluated over long periods of time (i.e., 3–5 y), is the secular (nonintervention-related) trends that may occur in both control and intervention groups because of substantial changes in underlying determinants of nutrition, such as income, food security, education, health services, or water and sanitation. Although difference-in-difference estimation is often used to net out the effects of common trends across control and intervention groups (7), statistically significant common trends should be analyzed and understood in their own right to inform policymakers about which nonintervention factors may be driving nutritional change in their targeted communities. This type of analysis could generate useful information for redesigning programs and strategies aimed at improving nutrition nationally and foster multisectoral collaboration toward the overarching goal of improving nutrition.

We showed in this study how the dynamic nature of program evaluation data collected across multiple rounds can be used to examine the extent to which different factors can statistically explain nutritional change over time. We used data from surveys collected in Bangladesh and Vietnam under the Alive & Thrive (A&T)^7^ program. A&T is an initiative to save lives, prevent illness, and contribute to healthy growth and development by improving infant and young child feeding (ICYF) practices (8). In both countries, the program was evaluated with the use of a cluster-randomized impact evaluation design with repeated cross-sectional surveys to measure changes in the main impact indicators (9). Two groups were compared: intensive (intensified interpersonal counseling + mass media + community mobilization) and nonintensive (standard nutrition counseling + less-intensive mass media and community mobilization). Difference-in-difference estimates found no statistically significant impacts of the intensive A&T interventions on child linear growth but showed rapid and statistically significant reductions in stunting in both intensive and nonintensive groups in each country (10, 11). In light of this rapid but unexplained change, we sought to understand what changes in underlying socioeconomic characteristics and behavioral factors are most important in explaining improvements in child linear growth in Bangladesh and Vietnam between 2010 and 2014.

## Methods

### Data sources

This study used data from the baseline (2010) and endline (2014) surveys of the A&T impact evaluation studies. These surveys were repeated cross-sections that tracked the sample survey clusters (villages) with the use of the same sampling strategy but different cohorts of children. This analysis followed the original sampling design (9) and focused on children aged 24–47.9 mo in Bangladesh (*n* = 4376) and 24–59.9 mo in Vietnam (*n* = 4012). Children in these age groups were potentially exposed to the A&T program during the entire first 1000 d (from pregnancy to 24 mo of age), the critical window of opportunity for improving nutrition. The sample of children aged <24 mo (*n* = 4189 in Bangladesh and *n* = 4054 in Vietnam) was also used to describe the patterns of linear growth from birth to 5 y of age in each survey round.

### Variables

The variables used in this analysis can be partitioned into outcomes, socioeconomic determinants of nutrition that were not explicitly targeted by the intervention, and the behavioral indicators that were targeted by the intervention.

### Outcomes

The outcomes in this analysis were child height-for-age *z* scores (HAZs) and height-for-age differences (HADs). The standing height of the children was measured in centimeters by a standardized method (12) with the use of locally manufactured collapsible height boards that were precise to 1 mm. Height was then converted into an HAZ according to 2006 WHO child growth standards (13). The HAD was calculated by subtracting the sex- and age-specific WHO 2006 growth standards median height (14) from the child’s actual height.

#### Socioeconomic determinants of nutrition not targeted by the intervention.

The underlying socioeconomic characteristics were selected based on the conceptual framework outlining the direct, underlying, and basic determinants of nutrition (6, 15) at various levels (household, maternal, and child factors). Household-level variables included the number of children aged <5 y, socioeconomic status (SES), food security, and exposure to an economic shock. The SES index was constructed with the use of principal component analysis of several variables such as house and land ownership, household assets, and access to services (16, 17). The first component was then rescaled to vary between 0 and 10, where higher scores indicated wealthier participants. Household food security was measured with the use of the Food and Nutrition Technical Assistance Project/US Agency for International Development Household Food Insecurity Access Scale (18); the scale was then divided into food-secure and food-insecure groups (including mild, moderate, and severely insecure). Economic shocks were measured based on maternal reports of any adverse events in the 12 mo before the survey, such as loss of employment of any household member, loss of crops, damage to the houses or productive assets, experience of natural disasters, and other shocks. Maternal measures included education, occupation, stress, height, weight, and BMI (in kg/m^2^). Maternal stress was measured with the use of Self-Reporting Questionnaire 20, which includes 20 items with a recall period of 30 d before the administration of the questionnaire (19). Child factors included age, sex, birth weight, and maternal recall of symptoms of diarrhea (≥3 loose stools passed in 24 h), as well as fever and cough or cold in the 2 wk before the survey (defined as acute respiratory infection).

#### Behavioral indicators potentially influenced by the intervention.

The socioeconomic indicators described previously were not expected to be influenced by the intervention, but data were also collected on a range of behavioral indicators that were either targeted by the A&T intervention and/or could have been influenced by the broader socioeconomic improvements observed in these 2 countries. These behavioral indicators include maternal knowledge of IYCF, child dietary diversity, use of prenatal services, and hygiene outcomes. Maternal knowledge of IYCF was assessed based on the mother’s answers to a series of questions related to breastfeeding and complementary feeding practices. Each knowledge item was given a score of 1 (correct) or 0 (incorrect), and the sum was used as the knowledge score (range: 0–8). Child dietary diversity was constructed based on the sum score (range: 0–7) of the children’s consumption of foods from 7 food groups in the past 24 h based on the WHO definition (20). Prenatal service use was categorized into 2 groups: <4 times and ≥4 times. Hygiene was assessed with the use of spot-check observations of the cleanliness of the mother and her child (hair, hands, faces, and clothing), as well as the cleanliness of areas inside and outside the household, a method that has been used widely for assessing markers of hygiene practices (21). Each hygiene item was given a score of 1 (clean) or 0 (dusty or dirty), and the sum was used as the hygiene score (range: 0–10).

### Data analysis

Statistical analyses were implemented for each country separately with the use of Stata version 13.1 (StataCorp). Descriptive analyses were first used to describe independent variables. Similar to well-known analyses of growth-faltering patterns in developing countries (22, 23), the HAZ and HAD distributions were graphed based on age with the use of a local polynomial smoother.

The differences for various determinant factors between 2 time points (baseline and endline) were estimated and tested for statistical differences with the use of linear regression models for continuous variables or logistic regression models for categorical variables to account for geographic clustering (24). This analysis indirectly indicated which variables might explain nutritional changes over time because indicators that did not change across rounds cannot explain any nutritional changes over time.

Multiple regression analyses were then conducted to examine the underlying determinants of HAZs and HADs. Because the behavioral factors might have been influenced by both the intervention and underlying factors, we estimated separate models for socioeconomic characteristics first, models for behavioral factors next, and combined models last. Household economic shocks, maternal stress, child acute respiratory infection, and diarrhea were not significantly associated with child HAZs or HADs and did not improve model fit; therefore, these variables were not selected in the final models. All models accounted for geographic clustering (24) and exposure to the intervention.

The last stage of our analysis—statistical decompositions of HAZ or HAD change over time—effectively combined the analysis of changes in means of the explanatory variables and regression estimates of the coefficients associated with these variables. In this technique, the “explained” difference between any 2 survey samples (e.g., the 2010 and 2014 A&T samples) accounted for by a particular variable (*X*) is the product of the difference in the mean of *X* across the 2 samples and the coefficient of *X* from a pooled regression model (Β_X_). It is intuitive that if a particular variable has a large regression coefficient (marginal effect) and a large change in its mean over time then this variable will play a large role in explaining changes in the dependent variable over time. One caveat is that the coefficient attached to *X* (Β_X_) may also change over time (e.g., the marginal effects of prenatal care on HAZs may change over time if the quality of prenatal care improves over time). The Oaxaca-Blinder technique addresses this problem by flexibly decomposing estimated changes in nutrition into the explained component described previously and unexplained component consisting of the product of any changes in coefficients and the pooled means. This approach has been used widely to study mean outcome differences between groups (25), including differences in child malnutrition between rural and urban areas (26, 27) and between populations measured at different time points (28). The decomposition analysis was applied to both HAZs and HADs, but only the results with HAZs are presented herein because our analysis of HADs resulted in similar results.

## Results

**FIGURE 1:**
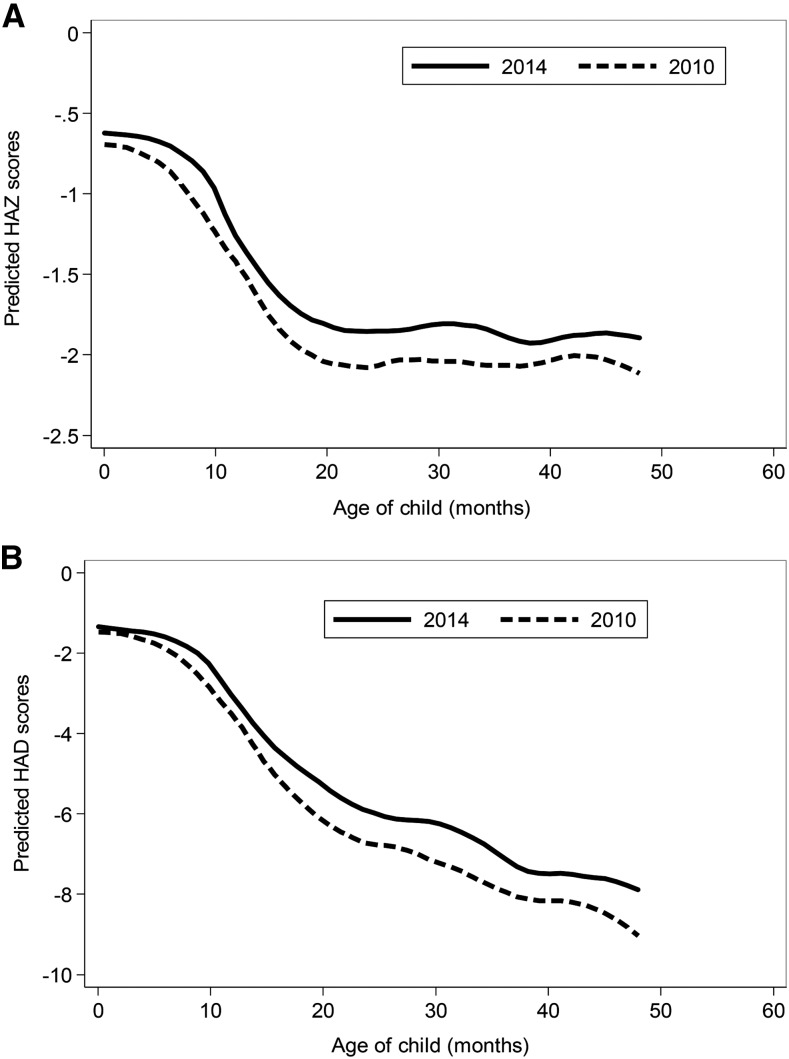
HAZ (A) and HAD (B) by age in children who participated in the 2010 and 2014 surveys from the Alive & Thrive impact evaluation in Bangladesh, *n* = 4189 children aged 0–23.9 mo and *n* = 4376 children aged 24–59.9 mo, respectively. HAD, height-for-age difference; HAZ, height-for-age *z* score.

Children in Bangladesh were born with a low HAZ (∼−0.7), but there were also rapid declines in HAZs from ∼4 to 20 mo of age, followed by growth stabilization (**Figure 1**). At endline, there were improvements in HAZs for all ages (0.18 SDs), with slightly larger changes for children aged >20 mo. Similar to HAZs, the HAD curves showed a mean absolute height deficit at birth of ∼−2 cm. The growth faltering started from 4 to 6 mo of age and continued after 24 mo of age. Figure 1 shows the continuous deterioration in HADs with the steep slope for all ages.

**FIGURE 2:**
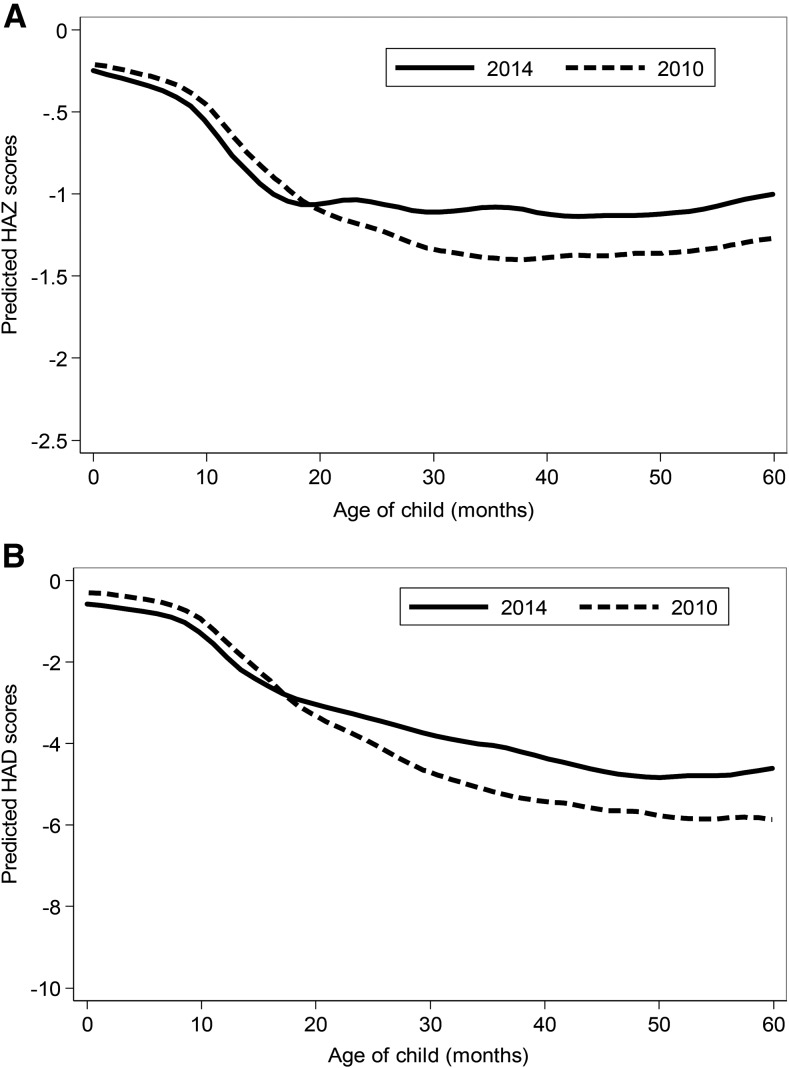
HAZ (A) and HAD (B) by age in children who participated in the 2010 and 2014 surveys from the Alive & Thrive impact evaluation in Vietnam, *n* = 4054 children aged 0–23.9 mo and *n* = 4012 children aged 24–59.9 mo, respectively. HAD, height-for-age difference; HAZ, height-for-age *z* score.

Vietnamese children were born with higher HAZs (∼−0.2) than Bangladeshi infants and showed less postnatal growth faltering (**Figure 2**). HAZs reduced sharply from ∼6 mo, continued in the first 20 mo, and then stabilized. HAZs improved significantly between 2010 and 2014 (0.25 SDs), but the significant improvements in HAZs were only observed for children aged >20 mo. Unlike the HAZs that stabilized after 20 mo, the HADs continued to drop until ∼50 mo of age.

In 2010, >50% and ∼25% of the children in Bangladesh and Vietnam were stunted, respectively. Stunting was substantially lower in both countries in 2014 (5.6% and 6.1%, respectively).

There were several important economic and social changes between 2010 and 2014 in both countries (**Table 1**). Specifically, household SES and food security improved substantially, although SES improved relatively more in Vietnam (38.9%) than in Bangladesh (13.6%). Women’s education improved almost equally in both countries; likewise, fewer women in both countries reported being a housewife or farmer as their primary occupation. Maternal health improvements were also observed in both countries, particularly in terms of reduced maternal stress and increases in weight and BMI scores (but not height). Child morbidity prevalence decreased, and child birth weight (or perception of birth weight in the case of Bangladesh) increased. We observed significant improvements in maternal IYCF knowledge, child dietary diversity, prenatal visits, and hygiene scores among the behavioral factors, although these improvements were substantially larger in Vietnam than in Bangladesh. For example, the percentage of mothers who received ≥4 prenatal visits increased by 49% in Vietnam, which was ∼50% greater than the improvement in Bangladesh (25%). Likewise, the improvement in hygiene scores was >3 times larger in Vietnam (1.98 points) than in Bangladesh (0.60 points), although the A&T program in Bangladesh involved some basic hygiene messaging.

**TABLE 1:** Selected characteristics of the study sample in the 2010 and 2014 surveys from the Alive & Thrive impact evaluation in Bangladesh and Vietnam^1^

	Bangladesh			Vietnam		
Characteristics	2010(*n* = 2177)	2014(*n* = 2199)	2010–2014	2010(*n* = 1968)	2014(*n* = 2044)	2010–2014
Household						
Children aged <5 y, *n*	1.08 ± 0.28	1.07 ± 0.26	−0.01 ± 0.01	1.18 ± 0.39	1.14 ± 0.37	−0.04 ± 0.01***
SES index score	5.06 ± 2.82	5.75 ± 2.78	0.69 ± 0.08***	4.45 ± 2.80	6.18 ± 2.71	1.73 ± 0.09***
Food insecurity, %	32.9	20.2	−12.7 ± 1.3***	41.8	28.3	−13.4 ± 1.49***
Economic shock score	0.20 ± 0.43	0.23 ± 0.50	0.03 ± 0.01*	0.80 ± 0.93	1.06 ± 1.05	0.26 ± 0.03***
Maternal						
Education, y	4.58 ± 3.59	5.42 ± 3.43	0.84 ± 0.11***	8.39 ± 3.00	9.27 ± 3.00	0.88 ± 0.09***
Farmers, %	92.4	74.8	−17.5 ± 1.09***	58.9	40.5	−18.5 ± 1.55***
Maternal stress, %	48.7	34.6	−14.1 ± 1.48***	34.5	26.8	−7.69 ± 1.45***
Weight, kg	46.9 ± 8.30	48.8 ± 8.67	1.89 ± 0.26***	47.1 ± 6.66	49.2 ± 7.32	2.07 ± 0.22***
Height, cm	151 ± 5.50	151 ± 5.42	0.16 ± 0.17	153 ± 5.21	153 ± 5.19	−0.17 ± 0.16
BMI, kg/m^2^	20.6 ± 3.30	21.4 ± 3.46	0.79 ± 0.10***	20.1 ± 2.47	21.0 ± 2.84	0.95 ± 0.08***
Children						
Boys, %	53.0	49.5	−3.53 ± 1.51*	52.2	55.4	3.15 ± 1.57*
Birth weight, kg	2.91 ± 0.89	3.00 ± 0.81	0.09 ± 0.03***	3.09 ± 0.47	3.15 ± 0.45	0.06 ± 0.02***
Age, mo	35.0 ± 6.65	35.1 ± 6.83	0.12 ± 0.20	40.7 ± 9.69	40.8 ± 10.2	0.11 ± 0.32
ARI, %	50.1	35.1	−15.1 ± 1.48***	40.9	29.5	−11.5 ± 1.50***
Diarrhea, %	5.93	3.91	−2.02 ± 0.65***	6.59	2.94	−3.65 ± 0.67***
Behavioral						
Maternal knowledge of IYCF score	3.65 ± 1.59	4.70 ± 1.55	1.05 ± 0.05***	3.71 ± 1.83	5.39 ± 1.96	1.69 ± 0.06***
Child dietary diversity score	3.82 ± 1.24	4.17 ± 1.42	0.35 ± 0.04***	4.99 ± 1.25	5.43 ± 1.07	0.44 ± 0.04***
Prenatal visit (≥4 times), %	23.3	48.3	25.0 ± 1.40***	24.5	73.8	49.3 ± 1.37***
Hygiene score	7.17 ± 3.05	7.77 ± 2.95	0.60 ± 0.09***	6.66 ± 2.47	8.64 ± 2.03	1.98 ± 0.07***

1 All values are means ± SDs for 2010 and 2014 unless otherwise indicated and means ± SEs for 2010–2014. *,***Significant change from baseline to endline: ***P < 0.001, *P < 0.05. ARI, acute respiratory infection; IYCF, infant and young child feeding; SES, socioeconomic status.

In the model of the multivariate regression analyses that only included socioeconomic characteristics (model 1) (**Table 2**), we found a significant relation between household SES scores and HAZs: each 1-point increase in the SES index (range: 1–10) was associated with a 0.04 SD increase in HAZs in both Bangladesh and Vietnam. In other words, the predicted HAZ difference between a child in the poorest and richest households in our sample was 0.4 SDs. Household food insecurity was strongly associated with child HAZs in Bangladesh (β = −0.16), but the association was small and only marginally significant in Vietnam. For maternal education, each additional year of schooling predicted a 0.02 SD higher HAZ, meaning that a household in which a mother who completed high school could be expected to have a child ∼0.2 SDs taller than a child born to a mother with no schooling. Maternal height was significantly associated with child HAZs in both countries and with similar magnitudes (β = 0.055 for Bangladesh and 0.056 for Vietnam). Child birth weight was associated with HAZs in both countries.

**TABLE 2:** Association between socioeconomic characteristics and behavioral factors with HAZs in Bangladesh and Vietnam^1^

	Bangladesh			Vietnam		
Characteristics	Model 1	Model 2	Model 3	Model 1	Model 2	Model 3
Age	−0.01 (−0.01, 0.00)*	−0.01 (−0.01, 0.00)	−0.01 (−0.01, 0.00)*	0.00 (−0.01, 0.01)	0.00 (−0.01, 0.01)	0.00 (−0.01, 0.01)
Boys	0.02 (−0.04, 0.09)	−0.01 (−0.08, 0.07)	0.01 (−0.06, 0.08)	0.00 (−0.06, 0.06)	0.06 (0.01, 0.12)*	0.01 (−0.05, 0.06)
Year (2014)	0.09 (−0.00, 0.19)^†^	0.08 (−0.01, 0.17)^†^	0.08 (−0.01, 0.18)^†^	0.14 (0.05, 0.24)**	−0.02 (−0.11, 0.06)	0.07 (−0.04, 0.17)
Program intervention	−0.03 (−0.13, 0.06)	−0.01 (−0.12, 0.10)	0.00 (−0.09, 0.09)	−0.03 (−0.14, 0.08)	−0.09 (−0.21, 0.04)	−0.04 (0.14, 0.07)
Program × year	−0.03 (−0.18, 0.12)	−0.11 (−0.27, 0.05)	−0.12 (−0.28, 0.04)	0.02 (−0.12, 0.16)	0.08 (−0.08, 0.23)	0.03 (−0.11, 0.17)
Children aged <5 y	−0.31 (−0.46–0.17)***	—	−0.29 (−0.43, −0.15)***	−0.17 (−0.27, −0.08)**	—	−0.17 (−0.26, −0.07)**
SES index	0.05 (0.03, 0.06)***	—	0.04 (0.02, 0.05)***	0.05 (0.03, 0.06)***	—	0.04 (0.03, 0.06)***
Household food insecurity	−0.16 (−0.25, −0.07)**	—	−0.11 (−0.20, −0.02*	−0.08 (−0.15, 0.00)^†^	—	−0.07 (−0.14, 0.01)^†^
Maternal education	0.03 (0.02, 0.04)***	—	0.02 (0.01, 0.03)**	0.02 (0.01, 0.03)**	—	0.02 (0.01, 0.03)**
Maternal height	0.06 (0.05, 0.07)***	—	0.05 (0.05, 0.06)***	0.06 (0.05, 0.06)***	—	0.06 (0.05, 0.06)***
Child birth weight	0.13 (0.08, 0.18)***	—	0.12 (0.07, 0.17)***	0.42 (0.35, 0.50)***	—	0.42 (0.34, 0.49)***
Hygiene	—	0.06 (0.06, 0.08)***	0.03 (0.02, 0.05)***	—	0.05 (0.04, 0.07)***	0.03 (0.01, 0.04)***
Prenatal visit (≥4 times)	—	0.03 (0.01, 0.05)*	0.01 (−0.01, 0.02)	—	0.17 (0.11, 0.24)***	0.08 (0.01, 0.14)*
Knowledge of IYCF	—	0.05 (0.03, 0.08)***	0.03 (0.01, 0.06)*	—	0.02 (0.00, 0.04)*	0.00 (−0.02, 0.02)
Child dietary diversity	—	0.08 (0.04, 0.12)***	0.04 (−0.00, 0.07)^†^	—	0.02 (−0.00, 0.05)^†^	−0.03 (−0.05, 0.01)
*R*^2^	0.17	0.09	0.19	0.23	0.07	0.24
*n*	4282	4311	4282	3699	4002	3699

1 All values are βs (95% CIs) unless otherwise indicated. Model 1 included underlying socioeconomic characteristics, child age, sex, time, program intervention, and interaction between time and program intervention; model 2 included behavioral factors and all other variables included in model 1; and model 3 included socioeconomic characteristics, behavioral factors, and all other variables included in model 1. ^†,^*,**,***Significant differences: ***P < 0.001, **P < 0.01,*P < 0.05, ^†^*P* < 0.10. HAZ, height-for-age *z* score; IYCF, infant and young child feeding; SES, socioeconomic status.

In model 2, which only included behavioral factors, hygiene scores (range: 0–10) had large and statistically significant coefficients with HAZs (β = 0.060 for Bangladesh and 0.054 for Vietnam); this means that the predicted HAZ difference between a child in the cleanest and least clean households were 0.60 and 0.54 SDs for Bangladesh and Vietnam, respectively. Having ≥4 prenatal visit yielded a much larger coefficient with HAZs in Vietnam (β = 0.172) than in Bangladesh (β = 0.026), perhaps suggesting large differences in the quality of services available. Knowledge of IYCF practices and child dietary diversity were also associated with HAZs in both countries. In the final model, which combined both socioeconomic characteristics and behavioral factors, the coefficients of socioeconomic variables remained stable, but the coefficients of behavioral variables reduced significantly and in some cases were no longer significant (such as prenatal visits in Bangladesh or knowledge of appropriate IYCF practices and child dietary diversity in Vietnam). This result is consistent with socioeconomic variables being underlying determinants of HAZs through changes in behavioral indicators.

The variables selected in the final regression models from Table 2 addressed the question of which factors significantly explained HAZs in Bangladesh and Vietnam. These variables were then used in the decomposition analysis to estimate the extent to which improvements in these factors contributed to changes in nutritional outcomes over time (**Table 3**). Table 3 reports the observed mean HAZs between 2010 and 2014 and the changes over time and divides these changes into 2 parts, one caused by changes in the mean values of the determinants between 2010 and 2014 (the explained component) and another by changes in regression coefficients and interactions between changes in coefficients and changes in means (the unexplained component). These results suggest that the changes in mean HAZs were primarily explained by changes in the means of the explanatory variables (i.e., the underlying determinants), with the model accounting for just over 80% of the change in HAZs in Bangladesh and 75% of those in Vietnam.

**TABLE 3:** Summary results for the Oaxaca-Blinder decomposition of HAZ changes in children between 2010 and 2014 in Bangladesh and Vietnam^1^

	Bangladesh	Vietnam
HAZ at baseline	−2.04 (−2.15, −1.92)	−1.35 (−1.44, −1.26)
HAZ at endline	−1.86 (−1.99, −1.72)	−1.09 (−1.16, −1.02)
HAZ change from baseline to endline	0.18 (0.10, 0.26)	0.25 (0.18, 0.33)
HAZ change accounted for by explanatory variables (explained)^2^	0.14	0.19
HAZ change accounted for by coefficients (unexplained)^3^	0.04	0.06
Share of HAZ change explained by the model, %	80.9	75.6
*n*	4282	3699

1 Values are βs (95% CIs) unless otherwise indicated. The decomposition is based on model 3 from Table 2, which includes both socioeconomic characteristics and behavioral factors plus other covariates and intervention group. HAZ, height-for-age *z* score.

2 The explained component refers to changes in nutrition outcomes accounted for by changes in the means of the explanatory variables multiplied by their corresponding regression coefficients from Table 2.

3 The unexplained component consists of 2 subcomponents: changes in coefficients across rounds and the interaction between changes in coefficients and changes in explanatory variables.

Regarding the individual contributions of changes in the means of the explanatory variables to changes in mean HAZs between 2010 and 2014, improvements in household SES and food security jointly contributed to a 0.04 SD increase in HAZs in Bangladesh, accounting for ∼21% of the total change in HAZs (**Table 4**). The contribution of these factors was even larger in Vietnam (although primarily of SES specifically), accounting for ∼33 of the HAZ changes. In both countries, increases in a mother’s educational attainment was a moderately strong predictor of HAZ change, although it is striking that education status improved so substantially in just a 4-y period. Improvements in birth weight made a similar contribution in both countries. Among the behavioral factors, improved hygiene scores explained 19% of the HAZ changes in Vietnam but just 10% in Bangladesh. Improved access to prenatal care was a significant contributor in Vietnam (25% of HAZ changes), but this factor was not significant in Bangladesh. In contrast, improvements in the knowledge of IYCF significantly contributed to HAZ changes in Bangladesh (20% of HAZ changes) but not in Vietnam.

**TABLE 4:** Disaggregated results for the Oaxaca-Blinder decomposition of HAZ changes between 2010 and 2014 in Bangladesh and Vietnam^1^

	Bangladesh		Vietnam	
	Change in HAZ from 2010 to 2014	Share of total change, %	Change in HAZ from 2010 to 2014	Share of total change, %
Children aged <5 y	0.01 (−0.01, 0.01)	1.80	−0.01 (−0.02, −0.01)**	0.00
SES index	0.03 (0.01, 0.04)***	12.4	0.07 (0.04, 0.09)***	26.3
Household food insecurity	0.02 (0.00, 0.03)*	9.07	0.01 (−0.01, 0.02)	2.53
Maternal education	0.02 (0.01, 0.03)**	12.9	0.02 (0.01, 0.04)*	7.38
Child birth weight	0.01 (0.00, 0.02)^†^	5.24	0.03 (0.01, 0.04)***	9.77
Hygiene	0.02 (0.00, 0.04)*	10.0	0.05 (0.01, 0.09)*	19.4
Prenatal visit (≥4 times)	0.01 (−0.01, 0.03)	4.39	0.06 (0.02, 0.10)**	24.7
Knowledge of IYCF	0.04 (0.00, 0.06)*	20.0	0.00 (−0.03, 0.03)	0.92
Child dietary diversity	0.01 (0.00, 0.03)^†^	0.85	−0.02 (−0.03, 0.01)	0.00

1 Values are βs (95% CIs) unless otherwise indicated. The predicted change in HAZs caused by changes in means multiplied by their respective coefficients from model 3 from Table 2 is shown. The decomposition is based on model 3 from Table 2, which includes both socioeconomic characteristics and behavioral factors plus other covariates and intervention group. ^†,^*,**,***Significant differences: ***P < 0.001, **P < 0.01, *P < 0.05, ^†^*P* < 0.10. HAZ, height-for-age *z* score; IYCF, infant and young child feeding; SES, socioeconomic status.

## Discussion

In the context of an experimental evaluation of A&T in which there was considerable improvements in HAZs in both intensive and nonintensive groups, we have proposed the application of a regression decomposition technique to identify which factors explained changes in HAZs over time in Bangladesh and Vietnam. Although several recent studies used this technique to explore drivers of nutritional change with nationally representative data (29–31), our study is the first to our knowledge to apply this technique to the evaluation of program data. This technique successfully explained HAZ changes over time, with the models accounting for 81% of the change in Bangladesh and 75% in Vietnam.

In Bangladesh, improvements in household SES, maternal education, and hygiene explained improvements in HAZs; these results are consistent with those from previous studies that used nationally representative data for Bangladesh from 1996 to 2011 (29), as well as multicountry assessments of the relation between economic indicators and child growth (28, 32, 33) and parental education and child growth (28, 34, 35). Unlike the earlier national-level study of nutritional change in Bangladesh (29), however, our study has also shown that improvements in food security, birth weight, and IYCF knowledge explain HAZ improvements in the A&T study areas. Data on these determinants were not available in the data set used for the earlier study, and the hygiene indicator was less specific (toilet use and improved water sources) than in this study, in which we used a more comprehensive measure of hygiene based on spot checks of the child, mother, house, and compound (29). Our study did not identify the prenatal care visit as an important driver of changes in HAZs in Bangladesh despite the fact that the use of prenatal care improved substantially between 2010 and 2014. In contrast, the prenatal care visit was a reasonably important factor in the earlier nationally representative study of HAZ improvements in Bangladesh (29). This might imply that interventions to improve child nutrition should focus more attention on improving the quality of prenatal care, especially because many Bangladeshi children are still born small and underweight, as our descriptive results have indicated.

To our knowledge, this is the first study to apply the regression decomposition technique to examine improvements in child nutrition outcomes in Vietnam. Our results for Vietnam shared some similarities to those of Bangladesh, although the magnitude of socioeconomic development in Vietnam seems to have been substantially larger. The mean annual economic growth rates in the 2 countries were similar (∼4.8%) (36), but improvements in SES were larger in the A&T survey areas in Vietnam than those in Bangladesh. The results from Vietnam also show more improvements in hygiene scores and access to prenatal care. Moreover, unlike Bangladesh, access to prenatal care was a reasonably strong predictor of HAZs in Vietnam. IYCF knowledge and child dietary diversity were not significant contributors to HAZ changes in Vietnam, perhaps because *1*) baseline dietary diversity was already high in Vietnam at baseline (88% of children aged 24–59 mo had consumed ≥4 food groups in the previous day), which left little room for improvement, and/or *2*) the quantity of food might be more limiting for child HAZs in Vietnam than diversity.

This study has several strengths but also some limitations. Previous studies have analyzed changes across nationally representative surveys in which it was safe to assume that changes in socioeconomic factors were not artifacts of any differences in survey design (29–31). The data used in this study were from repeated cross-sections from the same set of villages, but it is still possible that differences in the indicators across rounds stem from chance sampling differences rather than underlying socioeconomic trends. This study has 2 advantages, however. The first is the availability of a richer set of explanatory variables, such as hygiene and nutrition knowledge scores. The second is the application of a more flexible decomposition technique. Unlike previous studies (29–31) that used the decomposition-at-means approach, which assumes that the regression coefficients remain constant over time, our study used the flexible Oaxaca-Blinder technique, which allows for the instability of coefficients between rounds. The technique applied herein identified associations with regression models and assessed predictive power with the decomposition analysis. Neither step justifies claims to identifying causal relations. Hence, any inferences from this technique rest upon an assessment of plausibility and predictive power, not causality.

Although this caveat is important, program implementers do not have the luxury of making decisions based solely on experimental evidence, and understanding the specific drivers of undernutrition in a given context is critically important in designing successful programs. A challenge in applying the decomposition technique to program design is that drivers that were important in the past may not be important in the future and may therefore be imperfect in helping decision makers to select future investments. The current levels of underlying determinants and the potential to improve them must be considered in deciding what investments to make. Reducing undernutrition requires that many underlying determinants be at optimal levels, so the focus should be on determinants that are not at optimal levels at the time of making decisions about investments. The decomposition findings, however, can be useful for showcasing successes in improving nutrition in select countries or regions and a powerful way of illustrating the importance of collaboration among various sectors to achieve nutrition goals.

Our analyses strongly suggest that nonintervention socioeconomic factors were important predictors of nutritional change in both comparison groups and in both countries. In Vietnam, for example, improvements in SES, food security, maternal education, and hygiene explained >50% of the improvements in HAZs, yet none of these factors was specifically targeted by the A&T program. This result is consistent with the conclusion of the 2013 *Lancet* nutrition series that nutrition-specific interventions, if implemented at scale, would help tackle only 20% of the global burden of undernutrition (3) and that improvements in nutrition-sensitive programs from a variety of sectors are needed to accelerate progress in improving nutrition globally (15).

## Footnotes

7 Abbreviations used: A&T, Alive & Thrive; HAD, height-for-age difference; HAZ, height-for-age *z* score; IYCF, infant and young child feeding; SES, socioeconomic status.
